# Crimean-Congo haemorrhagic fever in a Greek worker returning from Bulgaria, June 2018

**DOI:** 10.2807/1560-7917.ES.2018.23.35.1800432

**Published:** 2018-08-30

**Authors:** Anna Papa, Filothei Markatou, Helena C. Maltezou, Elpida Papadopoulou, Eirini Terzi, Sarantoula Ventouri, Danai Pervanidou, Sotirios Tsiodras, Efstratios Maltezos

**Affiliations:** 1 National Reference Centre for Arboviruses and Haemorrhagic Fever viruses, Department of Microbiology, Medical School, Aristotle University of Thessaloniki, Thessaloniki, Greece; 2 Department of Internal Medicine, University Hospital of Alexandroupolis, Alexandroupolis, Greece; 3 Department for Interventions in Healthcare Facilities, Hellenic Center for Disease Control and Prevention, Athens, Greece; 4 Second Department of Internal Medicine, Medical School, Democritus University of Thrace, Alexandroupolis, Greece; 5 Department of Infection Control, University Hospital of Alexandroupolis, Greece; 6 Department for Epidemiological Surveillance and Intervention, Hellenic Center for Disease Control and Prevention, Athens, Greece

**Keywords:** Crimean-Congo haemorrhagic fever, imported, tick bite, Greece, Bulgaria

## Abstract

We report a tick-borne case of severe Crimean-Congo haemorrhagic fever (CCHF) imported into Greece from Bulgaria. The patient presented severe thrombocytopenia, hemophagocytosis, haemodynamic instability, large haematomas and altered mental status. Supportive treatment and ribavirin were administered. Symptoms started one day after the tick was removed; the patient was discharged from the hospital 26 days after symptom onset. No secondary cases were observed. Phylogenetically the CCHF virus strain belongs to clade Europe 1.

In June 2018, Crimean-Congo haemorrhagic fever (CCHF) was diagnosed in a Greek construction worker who returned home after becoming ill with fever and haemorrhagic symptoms in south-western Bulgaria. Here, we describe the case along with the epidemiological investigation and phylogenetic analysis.

## Case description

On 30 May 2018, a Greek male in his late 40s returned to Greece after spending 23 days in a forested area in Blagoevgrad province, south-western Bulgaria, where he was working in bridge construction. Three days earlier (27 May, day 1), while in Bulgaria, he developed fever, severe headache, myalgia (mainly in the lower extremities), malaise and loss of appetite; on 28 May he visited a local hospital and received symptomatic treatment as an outpatient. As his condition deteriorated (onset of photophobia and abdominal pain) he returned to his permanent residence in northern Greece. On 31 May (day 5), the patient was admitted to a local hospital. He was transferred to the university hospital in Alexandroupolis the next day because he presented severe thrombocytopenia and leukopenia; elevated levels of liver enzymes, creatine phosphokinase (CPK) and lactate dehydrogenase (LDH); and prolonged activated partial thromboplastin time (aPTT) (Table). On day 6, his headache was resolved but his fever (38.2 °C), malaise and myalgia were ongoing. The main laboratory findings were thrombocytopenia, prolonged aPTT (82 s) and increased level of aminotransferases. His laboratory parameters indicated rhabdomyolysis (CPK 1,739 U/L) and slightly elevated urea and creatinine levels (Table). A bone marrow biopsy showed haemophagocytosis.

**Table:** Serial haematological and biochemical parameters in a Crimean-Congo haemorrhagic fever patient, Greece, June 2018

Parameter (normal values)	Day of illness											
	5	6	7	9	10	12	14	15	17	19	22	24
WBC (x 10^9^/L) (3.50–10.80)	2.55	2.86	1.6	14.97	17.48	4.62	4.53	5.17	5.08	4.34	2.73	5.17
Neutrophils (%) (40–75)	69	46.6	30	80.2	78.6	48.2	62	72.3	73.6	58.5	43.9	43.3
Monocytes (%) (2–10)	1.2	5.2	10.6	5.6	10.2	19.9	14.8	12.6	9.1	9.7	11.4	6.6
Basophiles (%) (0.3–1.0)	0	2.8	5	1.7	1.7	1.3	0.4	0.4	0.8	1.8	2.9	0.4
Hematocrit (%) (35–45)	45.9	36.4	25.4	25.5	26.7	28.1	30.2	30.5	32.3	30.9	30.5	36.7
Platelets ( x 10^9^/L) (150–400)	10	21	22	31	31	28	35	53	78	137	146	139
aPTT (seconds) (25–37)	70.9	NA	NA	37.2	26.8	25.2	27.5	29	33,4	31.2	28	27.4
Fibrinogen (mg/dl) (220–490)	110.3	NA	NA	203	259	284	364	383	579	407	283	297
Urea (mg/dl) (20–50)	65	76	95	108	108	102	94	73	95	101	67	31
Creatinine (mg/dl) (0.6–1.1)	2.1	1.8	1.9	2.2	2	1.5	1.5	1.6	1.5	1.4	1.4	1
AST (U/L) ( < 33)	525	663	726	3,093	1,827	371	131	87	33	28	21	24
ALT (U/L) ( < 31)	116	150	166	870	638	257	144	103	60	39	26	34
LDH (U/L) 120–246	2,545	3,577	3,325	4,009	3,275	1,764	1,143	993	838	640	419	243
CK (U/L) (55–170)	1,479	1,739	1,343	474	357	204	119	92	52	38	36	NA
Total bilirubin (mg/dl) (0.3–1.2)	ΝΑ	NA	0.9	1.6	1.9	3.4	4.3	5.5	4	3.1	2	0,6
Direct bilirubin (mg/dl) ( < 0.2)	ΝΑ	NA	0.4	0.9	0.8	1.1	1.5	1.9	1.4	1.1	0,8	0,2
ALP (U/L) (30–120)	70	81	78	223	230	123	98	95	85	86	77	59
γ- GT (mg/dl) (7–32)	26	45	68	189	171	122	105	109	101	106	79	28
Serum amylase (U/L) (30–118)	ΝΑ	NA	272	205	175	130	169	207	178	140	NA	67

ALP: alkaline phosphatase; ALT: alanine aminotransferase; aPTT: activated partial thromboplastin time; AST: aspartate aminotransferase; CK: creatine kinase; LDH: lactate dehydrogenase; NA: not available; WBC: white blood cells; γGT: gamma-glutamyltranspeptidase.

**Figure 1:**
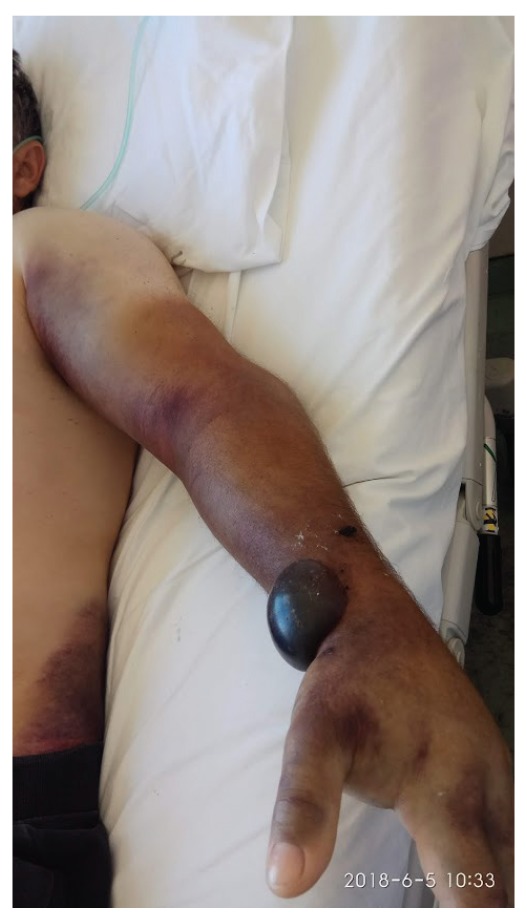
Large haematomas in a Crimean-Congo haemorrhagic fever patient (day 10 of illness), Greece, 5 June 2018

On day 6, the first day after admittance to the hospital in Alexandroupolis, the patient was asked about any recent tick bites; he mentioned that on 26 May he had found and removed a tick from his abdomen (he had not reported it to the hospital in Bulgaria or the first hospital in Greece). Rickettsiosis was suspected and treatment with oral vibramycin (100 mg x 2) was started. One day later (day 7), he presented with a progressively extended haematoma on his left upper arm (bleeding from venipuncture sites) and on his lower back (Figure 1). The patient’s clinical condition deteriorated rapidly, and on day 8 he presented with an abrupt drop in haematocrit, further elevation of transaminases and low fibrinogen (< 100 mg/dl), haemodynamic instability and altered mental status (lethargy). The laboratory findings in serial samples can be seen in Table. An abdominal computed tomography scan showed retroperitoneal haematoma and extensive haematomas in muscle groups at the site of bone marrow biopsy.

Based on the patient’s clinical presentation, and as he was bitten by a tick in an area of Bulgaria where CCHF cases have been reported previously, CCHF was highly suspected. Typically, the incubation period of CCHF after a tick bite is short (1–3 days), but the exact date of the bite was unknown in this case. The treating physician contacted the National Reference Centre for Arboviruses and Haemorrhagic Fever Viruses in Thessaloniki and the suspected case was immediately notified to the Hellenic Center for Disease Control and Prevention (HCDCP).

## Laboratory diagnosis

**Figure 2:**
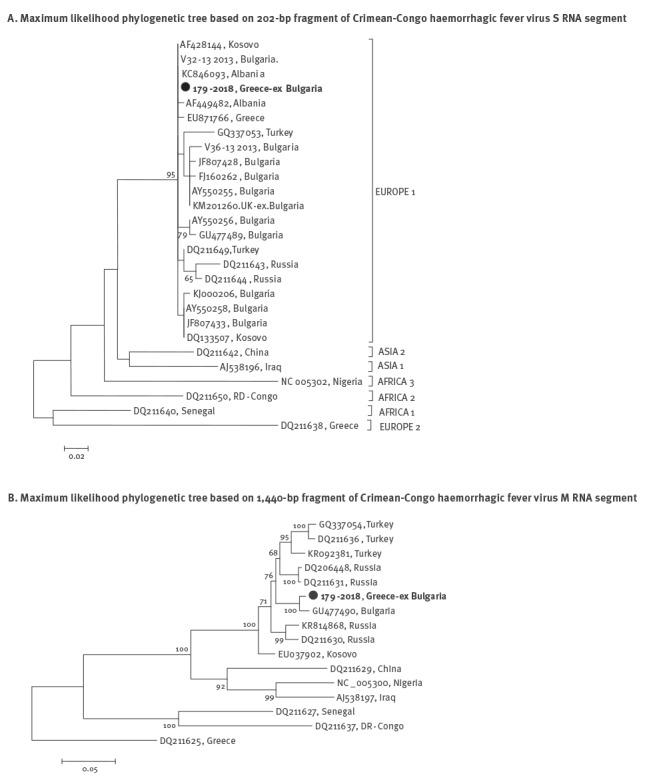
Maximum likelihood phylogenetic trees based on (A) 202-bp fragment of Crimean-Congo haemorrhagic fever virus S RNA segment and (B) 1,440-bp fragment of Crimean-Congo haemorrhagic fever virus M RNA segment, Greece, 2018

Blood and serum samples taken from the case on day 9 of illness were sent to the National Reference Centre. CCHF was laboratory diagnosed by serology and PCR 3.5 hours from the time of the sample receipt. CCHF virus (CCHFV) IgM antibodies were detected by ELISA (Vector Best, Novosibirsk, Russia); IgG antibodies were not detectable. Viral RNA was extracted from the blood sample using the QIamp viral RNA mini kit (Qiagen, Hilden, Germany). A real-time PCR (RT-PCR) (RealStar CCHFV RT-PCR Kit by Altona Diagnostics, Germany) resulted positive, with Ct-value 24.22. The viral load measured by an additional RT-PCR (method previously described in [1]) was 3.33 X 10^7^ copies per ml of plasma, suggesting that it was still high on day 9 of illness. In order to obtain sequences, a nested RT-PCR that amplifies a partial fragment of the CCHFV S RNA segment was applied [2]. Additionally, a 1,440-bp fragment of the M RNA segment was amplified. The sequences of the S and M segments were aligned with respective sequences from the GenBank database using CLUSTAL W and phylogenetic analysis was performed using MEGA v.7 [3]. It was shown that the patient’s strain clustered in the CCHFV lineage Europe 1, together with other strains from the Balkan Peninsula (Figure 2).

The Kimura 2-parameter model was applied. The trees are drawn to scale, with branch lengths measured in the number of substitutions per site. The percentage of replicate trees in which the associated taxa clustered together in the bootstrap test (1,000 replicates) is shown next to the branches. Bootstrap values lower than 60% are not shown. Sequences in the trees are indicated as GenBank accession number and country of isolation. Sequences of the present study are marked.

## Treatment and outcome

Upon admission to the second hospital in Greece, transfusion with fresh frozen plasma was initiated (3 units) and continued for 11 days (2–10 units daily). The patient was also transfused with concentrated red blood cells (2–4 units daily) and platelets (5–10 units daily). Following the laboratory confirmation of CCHF and although 10 days had passed from the onset of illness, oral ribavirin was initiated with a starting dose of 1 g (glomerular filtration rate = 40 ml/min), followed by 500 mg x 4 for 3 additional days. On day 14, the patient’s condition improved and laboratory parameters gradually returned to normal levels, at which time ribavirin was discontinued. The patient was discharged on day 26. One week later he was re-evaluated (clinically and laboratory) and found in good condition. A serum sample tested 1 month after discharge was positive for CCHFV IgG and IgM antibodies and negative for viral RNA.

## Epidemiological investigation and interventions

The HCDCP investigated the case immediately after the diagnosis of CCHF (through telephone interviews with a close family member and with the patient, after recovery, to confirm the dates) and his contacts while he was ill (household members, co-workers, roommates in Bulgaria and relatives who visited him in the hospitals). Close contacts were tested for CCHF and monitored for 14 days for any symptom development. The risk for further transmission was also assessed. The HCDCP promptly informed the Bulgarian health authorities about the case; they also informed the patient’s Greek co-workers in Bulgaria about prevention and proper management of tick bites (informative material in Greek was sent to them) advising them to seek medical care in case they develop symptoms. No other cases were reported among the patient’s co-workers in Bulgaria, up to the end of July 2018. The regional and local public health authorities were also informed about the case and they performed further contact investigation in Greece. No secondary cases were detected. The HCDCP raised awareness for CCHF among health professionals working in local health centres and hospitals in northern Greece, especially in areas with populations travelling to Bulgaria for occupational reasons.

## Infection control

The patient and his laboratory samples, apparel, waste and cleaning procedures were managed in accordance with the national guidelines for viral haemorrhagic fevers (available in Greek from HCDCP website: http://www.keelpno.gr/). In particular, upon the suspicion of CCHF (day 8) the patient was immediately isolated and strict barrier precautions were utilised (waterproof gowns, gloves, FFP3 respiratory masks, goggles), and personal protective equipment was used by healthcare workers (HCWs) and visitors; however, visitors were discouraged from entering the isolation room. The HCDCP sent guidelines for contact tracing and active surveillance of symptoms in HCWs possibly exposed to CCHFV. Patients who were hospitalised in the same room with the patient before the suspicion of CCHF (two patients in the first hospital (days 5–6), and three patients in the second hospital (days 6–8)), were also monitored for symptoms for 14 days after their last contact with the patient. No secondary cases were observed.

## Discussion

Crimean-Congo haemorrhagic fever (genus Orthonairovirus, family *Nairoviridae*) circulates in nature in an enzootic cycle between ticks and non-human vertebrates. It is transmitted to humans by bite of infected Ixodid ticks (mainly *Hyalomma* spp.) or by contact with blood or tissues of viraemic patients or animals [4]. Nosocomial infections are often reported. Here we report a CCHF case imported into Greece from south-western Bulgaria, where the patient was working on a construction site. He presented with the first symptoms 1 day after the removal of a tick from his abdomen.

CCHF was first recognised in Bulgaria in 1952; since then, several cases have been reported. Genetic characterisation of the Bulgarian strains showed that they cluster into the clade Europe 1 [5]. Our patient was infected in an area that was considered at low risk for CCHF outbreaks up to 2008, when a cluster of cases was observed in the region [6]. Although the seroprevalence in the human population in Blagoevgrad province is low (1%) [5], a seroprevalence of 41.9% in livestock was reported recently [7]. Since CCHFV is transmitted mainly by bite of infected Ixodid ticks, persons living in rural areas are at increased risk for acquiring the infection. This was the reason that information about preventive measures was sent to our patient’s Greek co-workers in Bulgaria, and all related public health authorities were informed about the case.

Regarding Greece, no other imported cases have been reported so far and the only autochthonous CCHF case was observed in 2008 [8]. A review of travel-associated CCHF cases published during 1960–2016 reported 21 cases [9]; two imported cases have been reported within Europe: Bulgaria to Germany in 2001 [10] and Bulgaria to the United Kingdom in 2014 [11].

Due to the high pathogenicity of the CCHFV, the absence of a specific drug treatment or vaccine and the risk of person-to-person transmission, rapid diagnosis is crucial to ensure that appropriate infection control measures (e.g. isolation of patient and barrier precautions) can be implemented in a timely manner. A detailed medical history of the patient, including travel history and possible risk factors, is important for the timely diagnosis of the disease. In our case, information regarding the tick bite was not provided immediately and this, in combination with the non-specific initial symptoms, meant that CCHF was first suspected on day 8 of illness. Despite this delay, the patient fully recovered and no secondary cases of CCHF have been reported. Since the northern part of Greece is close to CCHF-endemic countries, HCWs in this region should be made aware of CCHF; including the provision of training to better help them address questions from patients about travel history (identify potential risk of exposure). Physicians should include CCHF in the differential diagnosis for patients with haemorrhagic syndromes, especially if patients report a tick bite, outdoor activities or occupation in rural areas and recent travel to an endemic area.
